# Failures in the case management of children with uncomplicated malaria in Bata district of Equatorial Guinea and associated factors

**DOI:** 10.1371/journal.pone.0220789

**Published:** 2019-08-02

**Authors:** Pablo Suárez-Sánchez, Belén García, Jesús Nzang, Policarpo Ncogo, Matilde Riloha, Pedro Berzosa, Agustín Benito, María Romay-Barja

**Affiliations:** 1 Centro Nacional de Medicina Tropical, Instituto de Salud Carlos III, Madrid, Spain; 2 Servicio de Medicina Preventiva, Hospital Clínico San Carlos, Madrid, Spain; 3 Centro de Referencia de Control de Endemias, Malabo, Equatorial Guinea; 4 Ministerio de Salud y Bienestar Social, Malabo, Equatorial Guinea; 5 Red de Investigación Colaborativa en Enfermedades Tropicales, RICET, Madrid, Spain; Swiss Tropical & Public Health Institute, SWITZERLAND

## Abstract

**Background:**

In Equatorial Guinea, malaria continues to be one of the main causes of morbidity and mortality among children. The National Therapeutic Guide established artesunate-amodiaquine (ASAQ) as first-line treatment for uncomplicated malaria, but compliance with this treatment is low. The aim of this study was to assess, for the first time, the performance of public healthcare workers in the diagnosis and treatment of uncomplicated malaria, their compliance with first-line Malaria National Therapeutic Guide and the associated factors.

**Methods:**

A cross-sectional survey was conducted at the nine public health facilities in the Bata District of Equatorial Guinea to assess the management of uncomplicated malaria in children < 15 years of age. Bivariate and multivariate statistical analyses were used to determine the recommended treatment compliance and related factors.

**Results:**

A total of 227 children with uncomplicated malaria were recorded from 9 public health facilities. Most of the treatments prescribed (83.3%) did not follow the first-line treatment recommended for uncomplicated malaria. The diagnosis was established with parasite confirmation in 182 cases (80.2%). After adjustment for other variables, children under 2 months of age, the use of parasite confirmation to the diagnosis of malaria and being familiar with the national therapeutic guide were significantly associated with the prescription of the first-line recommended treatment. Cases attended at the hospital or in a health facility with ASAQ in the pharmacy at the time of the study were also more likely to be prescribed with the recommended treatment, but with non-significant association after adjustment for other variables.

**Conclusions:**

This study identified the factors associated with the low compliance with the first-line treatment by the public healthcare facilities of Bata District of Equatorial Guinea. It seems necessary to improve case management of children with uncomplicated malaria; to reinforce the use of Malaria National Therapeutic Guide and to inform about the danger of using artemisinin monotherapy. Furthermore, it is crucial to provide recommended first-line treatment to the pharmacies of all public health facilities to ensure access to this treatment.

## Introduction

Malaria continues to be one of the main causes of premature death in Africa. Children < 5 years of age are the most vulnerable group, accounting for 61% of all malaria deaths [1]. Prompt diagnosis and treatment is the most effective way to prevent malaria from developing into severe disease and death. To improve treatment efficacy and overcome drug resistance to former antimalarial drugs, such as chloroquine (CN) and sulfadoxine-pyrimethamine (SP), the World Health Organization (WHO) recommended the use of artemisinin-based combination therapy (ACT) as first-line treatment for uncomplicated malaria since 2001 [2].

Effective case management at public health facilities is one of the cornerstones of malaria control [3]. *Plasmodium falciparum* resistance to artemisinin and its derivatives has spread in Southeast Asia and is threatening malaria control efforts. In sub-Saharan Africa, low or inadequate use of artemisinin combination therapies (ACTs) may increase the malaria burden and the risk of widespread parasite resistance [4] as presumptive treatment, co-medication, low ACTs quality, and the use of artemisinin monotherapy persists [5]. Artemisinin monotherapy can contribute to the emergence of resistance to ACTs and lead to treatment failure [6].

Malaria is an endemic disease in Equatorial Guinea [7]. According to the WHO, there were an estimated 435,143 cases of malaria in 2017 in Equatorial Guinea, one of the countries with an increase in case incidence since 2010 [1]. Malaria continues to be one of the main causes of morbidity and mortality among children, and represents 21% of the causes of death in children < 5 years of age [8]. Furthermore, the percentage of malaria cases treated with antimalarial medications in Equatorial Guinea remains lower than many other countries in the region [1].

During recent years, national programs have adapted the WHO treatment recommendations into their local context. In Equatorial Guinea, the National Program to Malaria Control (NPMC) of the Ministry of Health and Social Welfare (MINSABS in Spanish) updated its Malaria National Therapeutic Guide (MNTG) in 2008, establishing ACT artesunate-amodiaquine (ASAQ) as first-line treatment for uncomplicated malaria. The use of oral artemisinin monotherapy was forbidden in Equatorial Guinea in 2014 [9]; however, nine years after the introduction of ASAQ therapy in Equatorial Guinea, compliance with the first-line treatment seems to be very poor in the Bata district. In previous studies, caregivers reported that injectable artemether (AM) monotherapy was used as first-line treatment for uncomplicated malaria in children in the Bata District of Equatorial Guinea, independent of where they sought treatment [10,11]. The inadequate use of artemisinin monotherapy involves serious risks leading to parasite resistance and disease recrudescence [12].

The compliance of healthcare workers with national treatment recommendations is fundamental for the success of malaria control policies but there are many problems for implementation at the healthcare provider level [13]. In many African countries, treatment compliance depends on a functional supply chain, beliefs established in the community, lack of knowledge regarding treatment guidelines and the existence of a broad alternative prescribing practice [14–18].

The aim of this study was to assess, for the first time, the diagnosis and prescription practices for uncomplicated malaria in the Bata District of the continental region in Equatorial Guinea. The degree of compliance with the Malaria National Therapeutic Guide (MNTG) and the associated factors were evaluated to facilitate design strategies addressed to reinforce malaria case management.

## Methods

### Study design and setting

The Bata District, in the Litoral province of the continental region of Equatorial Guinea, is the largest district of the country with a population of 309,345 inhabitants [7]. The proportion of the population living in urban areas increased from 27.1% in 1975 to 95.3% in 2015 [19].

The public health system structure in the Bata District consists of 10 public health facilities formed by 9 health centers and 1 regional hospital, this last one located in the city of Bata. At the time of this study, only 8 health centers were operational (2 rural and 6 urban). Despite the public status of the health institutions, in Bata District patients must pay for consultations, complementary tests, and treatments. Management of the public facilities are heterogeneous (government, NGOs, and religious congregations) with different prices, patient flow, and availability of drugs in stock. Private pharmacies and other pharmaceutical sellers are distributed throughout the rural and urban areas of the district. There are also some private health facilities in the Bata District, including two hospitals, and about 23 clinics, all in the urban area of Bata city. The last prevalence of malaria rate known among children < 15 years of age was 41.2% in 2013 [20].

The Equatorial Guinea MNTG was the reference for this study, where ASAQ is the first-line treatment recommended in all children > 2 months of age with malaria, and quinine (QN) is recommended for younger patients. Artemether-lumefantrine (AL) and QN remains second-line treatment when ASAQ is not effective. Intravenous artesunate is recommended for the management of severe malaria until the patient regains consciousness, then full ASAQ therapy should be administered. If artesunate is not available, quinine or artemether should be administered [9].

The NPMC has implemented a set of malaria control interventions in the last 20 years in Equatorial Guinea. A large-scale control program, the Equatorial Guinea Malaria Control Initiative (EGMCI), was launched in the continental region in 2007 to improve the malaria case management with the distribution of free ASAQ to all health facilities. The EGMCI ceased its activities in the region in 2011 due to a lack of funding and the National Program stopped then the supply of antimalarial drugs to the public health facilities of the continent [7,21]. This lack of governmental provision affected the pharmacies of all public health facilities in Bata, including the regional hospital. Since 2012, the pharmacy managers of the public health facilities have to purchase their own antimalarial drugs to the private distributors. From there on, each health facility is deciding which antimalarial to acquire, following their own criteria and without governmental supervision.

### Study population and data collection

A cross-sectional survey was conducted, between October and December 2017, in the Bata District of Equatorial Guinea to assess management of uncomplicated malaria in patients < 15 years of age by public healthcare practitioners. Health facility surveys and cases registration were carried out at all nine operative public health facilities. There were 28 physicians, 13 nurses and 3 medical assistants who attended patients in consultation in the Bata District. All health professionals who attended patients under 15 years old with suspected malaria were included in the study and followed up during one day of consultation. Data was systematically collected by two trained fieldworkers in three questionnaires. The first for the cases registration, the assessment of the health worker malaria diagnosis and treatment practices: registration of the characteristics of patients diagnosed with malaria (age, weight, days with fever, main signs and symptoms), the availability and use of malaria diagnostic tools, such as rapid diagnostic tests (RDTs) or microscopy. This information was systematically recorded from direct observation while accompanying each health practitioner during consultation. Measures were taken in order to minimize the Hawthorne effect [22]. The health workers observed were informed about the objective of the study and the anonymity of the data collected. Visits to the same health facilities were alternated in time to reduce the pressure on the health workers and avoid comparisons between them. Data collection for patients was also fully anonymous.

The second questionnaire was carried out for assessing the availability of ASAQ and any other antimalarial drugs at the pharmacy. At the end of the consultation day, a third questionnaire was answered by every health worker included in the study with questions about their sociodemographic characteristics, malaria diagnosis, their knowledge about the Malaria National Therapeutic Guide, which antimalarial they considered as the best treatment and other related questions. Data was doubly entered with EpiInfo 7.2 (CDC).

### Data analysis

Any healthcare worker who performed consultations and prescribed antimalarial medications to patients has been considered as a practitioner, irrespective of their professional background. Malaria management of children with suspected malaria was recorded, but only patients diagnosed with uncomplicated malaria were included in the analysis.

The variable “Recommended prescription for uncomplicated malaria”, was created according to first-line treatment of the Malaria National Treatment Guide based on the age of the patient, as follows: QN if the child was < 2 months of age; and ASAQ for children 2 months-to-15 years of age [9]. When the practitioner obtained a hemoglobin level, the results were categorized for analysis according to the WHO hemoglobin levels to diagnose anaemia at sea level [23]. Finally, within the third questionnaire, the prescribers were asked about whether they knew or not the Malaria National Treatment Guide and their answers are shown here in a variable labelled "Knowing the MNTG".

Frequencies and percentages were used to summarize data in the descriptive analysis. Mean and standard deviation or median and interquartile range were calculated for continuous variables that were or were not normally distributed, respectively. ÷2 tests were performed for bivariate analysis of the associations between recommended treatment and independent variables. The Fisher exact test was used when any expected count was <5. Comparisons for which p values were <0.05 were considered significant. A backward LR multivariate analysis was performed to identify associations between recommended treatment and independent variables with p values <0.10 on univariate analysis. Data analysis was performed using IBM SPSS statistics 23.

### Ethics statement

This study was approved by the Ministry of Health and Social Welfare of Equatorial Guinea and the Ethics Committee of the Spanish National Health Institute, Carlos III (CEI PI 71_2017). Written informed consent was obtained from all participants in the study.

## Results

Information involving 262 patients diagnosed with malaria was registered at the 8 operational public health centers and the regional hospital of Bata district, but 35 (12.2%) were diagnosed with complicated malaria. Therefore, a total of 227 patients with uncomplicated malaria were included in this study. Most of the malaria cases were children 1–5 years of age (55.1%), from the urban area (91.63%) and with no differences between sexes. High parasitemia was found in 12.6% of the blood films.

Regarding treatment, most prescriptions observed (83.3%) did not follow the first-line treatment MNTG recommended for uncomplicated malaria. Furthermore, 84.4% of the patients who were eligible to receive ASAQ, received other treatment (Table 1).

**Table 1:** Treatment administered by patient age.

	AS/AQ		AL		AS/MQ		AS		QN		AM		CN		Total
Age	n	%	n	%	n	%	n	%	n	%	n	%	n	%	n
< 2 months	0	0.00	2	28.57	0	0.00	0	0.00	3	42.86	0	0.00	2	28.57	7
2–11 months	1	2.78	20	55.56	0	0.00	0	0.00	6	16.67	9	25.00	0	0.00	36
1–5 years	27	21.60	45	36.00	1	0.80	10	8.00	0	0.00	42	33.60	0	0.00	125
5 to 15 years	7	11.86	16	27.12	0	0.00	4	6.78	0	0.00	32	54.24	0	0.00	59

AS/AQ: Artesunate-Amodiaquine; AL: Artemether-Lumenfantrine; AS/MQ: Artesunate-Mefloquine; AS:Artesunate; QN: Quinine; AM: Artemether; CN: Chloroquine

Atemether was the antimalarial most prescribed among children older than five years (54.2), while patients between 2 and 11 months mainly received the ACT artemether-lumefantrine (AL) as prescription (55.6%). Children <2 months were manly prescribed quinine (42.8%) but also chloroquine (28.6%). The prescription of sulfadoxine-pyrimethamine (SP) was recorded quite frequently (35.7%) but always as a complementary treatment. SP was prescribed in a single dose mainly with intramuscular AM (80.2%) or oral AS (14.8%).

### Patient-related factors

The symptoms of malaria patients were self-reported fever (94.3%), cough (47.6%), loss of appetite (22.9%), vomiting (22.0%), and weakness (19.8%). Clinical signs of anaemia and splenomegaly were only observed in 7.5% and 1.8% of the patients, respectively.

From 214 children reporting fever, 183 (85.5%) knew the onset day. Among those, only 44 (24.0%) sought treatment within the first 24 hours. The median duration of fever at the time of the consultation was 2 days (IQR: 2–3). Practitioners took the axillary temperature with a thermometer only in 83 cases (36.6%), presenting fever 46 (55.42%) of them. More than half of the children 154 (61.8%) were weighed. Hemoglobin levels were measured in 107 children (47.1%), with an average of 10.38 gr/dl (SD: 1.67); among those, 70 (65.4%) of the children were anaemic and 42.9% had moderate anaemia. Only the age of the patients was significantly associated with the treatment prescribed (Table 2).

**Table 2:** Characteristics of patients and symptons by prescription.

Characteristics of patients	Recommended prescription		Non-recommended prescription		P value
	n	%	n	%	
**Sex:**					
Female	12	12.12	87	87.88	
Male	26	20.31	102	79.69	0.101
**Age group:**					
<2 months	3	42.86	4	57.14	
2–11 months	1	2.78	35	97.22	
1–5	27	21.60	98	78.40	
>5	7	11.86	52	88.14	0.009
**Residence**					
Urban area	35	16.83	173	83.17	
Rural area	3	20.00	12	80.00	
Another district	0	0.00	4	100.00	0.631
**Clinical signs and symptoms**					
Fever	37	17.29	177	82.71	0.701
Cough	21	19.44	87	80.56	0.298
Loss of appetite	9	17.31	43	82.69	0.901
Vomits	5	10.00	45	90.00	0.148
Weakness	5	11.11	40	88.89	0.259
Anemia	5	29.41	12	70.59	0.172
Splenomegaly	2	50.00	2	50.00	0.131
Diarrhoea	6	25.00	18	75.00	0.253
Weight (n = 154)*					
<4.5 kg	0	0.00	2	100.00	
4.5–8 kg	0	0.00	23	100.00	
9–17 kg	17	20.48	66	79.52	
18–35 kg	5	13.89	31	86.11	
>35 kg	0	0.00	10	100.00	0.077
Haemoglobin levels (n = 107)*					
No anaemia	6	16.22	31	83.78	
Mild anaemia	2	8.70	21	91.30	
Moderate anaemia	9	19.57	37	80.43	
Severe anaemia	0	0.00	1	100.00	0.671

*Data of those in which the prescriber has measured this information

### Practitioners-related factors

A total of 41 practitioners were observed during medical consultations; 26 (63.4%) were at the health centers and 15 (36.6%) at the regional hospital. Most of the practitioners were women, <45 years of age (65.9%) with a degree career in medicine (57.7%) and a median experience of 3 years (IQR): 2–9.5) (Table 3). Practitioners established the diagnosis based on parasite confirmation for 182 patients (80.2%), 154 were confirmed by microscopy (84.6%) and the remainder by RDT. The diagnosis in 19.8% of the children prescribed with antimalarial drugs was presumptive, including 20 patients that had negative laboratory tests results.

**Table 3:** Compliance with the MNTG related to practitioner characteristics and practices.

	Recommended prescription		Non-recommended prescription		P value
	n	%	n	%	
**Sex:**					
Female	28	18.79	121	81.21	
Male	10	12.82	68	87.18	0.252
**Age group:**					
26–35	15	16.85	74	83.15	
36–45	7	8.33	77	91.67	
>45	16	29.63	38	70.37	0.005
**Finished studies:**					
Medicine degree	29	22.14	102	77.86	
Nursing degree	5	22.73	17	77.27	
Nursing diploma	3	5.17	55	94.83	
Medical assistant	1	6.25	15	93.75	0.017
**Years of experience**					
0–3 years	21	18.75	91	81.25	
>3 years	17	14.78	98	85.22	0.423
**Diagnosis based on:**					
Presumptive diagnosis	3	6.67	42	93.33	
Parasite confirmation	35	19.23	147	80.77	0.055
**MNTG knowledge**					
No	7	6.31	104	93.69	
Yes	31	26.72	85	73.28	<0.001

Older practitioners and practitioners with higher education were significantly more compliant with the treatment recommended. Compliance with the MNTG was also greater among those practitioners that made parasite confirmation for diagnosis and among those who knew the MNTG (52.3%). No significant differences were found by practitioners’ sex.

### Health facility-related factors

Regarding the characteristics of the health facilities, only two public health centers had first-line antimalarial treatment (ASAQ) in stock at the time of the study, and two health centers (one rural and one urban) did not have any malaria diagnostic tool (Table 4).

**Table 4:** Description of public healthcare facilities in the Bata District.

Health institution	Availability of diagnostic tool		ASAQ in stock**	Uncomplicated malaria cases visited during the period of the study
	Rapid Diagnostic Test	Working microscopy		
SOS Aldeas Infantiles	**+**	**+**	**-**	39
M.^a^ Gay	**+**	**+**	**+**	23
M.^a^ Rafols	**-**	**+**	**-**	40
La libertad	**-**	**+**	**-**	25
Ukomba	**+**	**+**	**+**	16
Río Campo*	**-**	**-**	**-**	2
Oyarregui	**-**	**-**	**-**	9
Machinda*	**+**	**+**	**-**	11
Regional Hospital	**-**	**+**	**-**	62
***Total***	***4***	***7***	***2***	***227***

*Rural Health Centers

**Available in pharmacy of the health institution at the moment of the study

There were no significant differences in compliance of the recommended malaria treatment regarding the type of health facility but ASAQ was more frequently prescribed at the regional hospital. Despite the fact that 69.2% of children consulted in health facilities with ASAQ in stock were not prescribed with this treatment, ASAQ was significantly more frequently prescribed in health facilities where the treatment was available (Table 5).

**Table 5:** Health facilities related factors.

	Recommended prescription		Non-recommended prescription		P value
	n	%	n	%	
**Clinic type**					
Regional hospital	15	24.19	47	75.81	
Health center	23	13.94	142	86.06	0.065
ASAQ in stock*					
No	26	13.83	162	86.17	
Yes	12	30.77	27	69.23	0.010

*Available in pharmacy of the health institution at the moment of the study

### Factors associated with MNTG treatment compliance

Based on multiple logistic regression analysis, children <2 months of age were 6 times more likely to be prescribed with the first-line recommended treatment. Regarding case management, those practitioners that used parasite confirmation to establish the diagnosis of malaria were almost 6 times more likely to prescribe recommended treatment, and the practitioners familiar with the MNTG were almost 3 times more likely to follow the MNTG. Practitioners working at the hospital or in a health facility with ASAQ in the pharmacy at the time of the study, were also more likely to prescribe the recommended treatment, but with a marginally non-significant association after adjustment for other variables (Table 6).

**Table 6:** Analysis of factors associated with recommended prescription according to MNTG.

	Unadjusted OR	(95% CI)	P value	Adjusted OR	(95% CI)	P value
**Patient age**						
< 2months	1			1		-
2 months—15 years	0.25	(0.05–1.18)	0.094	0.16	(0,03–0,95)	0.044
**Prescriber Age**						
26–35	1			1		0.058
36–45	0.45	(0.17–1.16)	0.099	0.63	(0,21–1,84)	0.396
>45	2.08	(0.93–4.65)	0.075	2.36	(0,85–6,54)	0.097
**Prescriber Studies**						
Other studies	1			-	-	-
Medicine degree	2.75	(1.23–6.12)	0.013	-	-	-
**Diagnosis based on**						
Presumptive diagnosis	1			1		
Parasite confirmation	3.33	(0.98–11.38)	0.055	5.56	(1,40–22,14)	0.015
**MNTG Knowledge**						
No	1			1		
Yes	5.42	(2,27–12,92)	<0,001	2.92	(1,12–7,63)	0.029
**Clinic type**						
Health Center	1			1		
Regional Hospital	1.97	(0.95–4.09)	0.068	2.55	(0,92–7,08)	0.072
ASAQ in stock*						
No	1			1		
Yes	2.77	(1.25–6.14)	0.012	2.72	(0,96–7,70)	0.059

*Available in pharmacy of the health institution at the moment of the study

## Discussion

Most uncomplicated malaria cases in the Bata District were diagnosed based on parasite confirmation, but prescribed without following the first-line treatment recommended in the MNTG. Low compliance with national protocols by healthcare workers remains a serious problem in many countries in sub-Saharan Africa [24,25].

This lack of compliance with the national protocol has been described in many other countries in the region [15,16,25,26]. However, the use of injectable AM as first-line of treatment for malaria is not common in the region. A similar frequent use of AM or injectable QN to treat children with uncomplicated malaria has only been described in Sudan [15,27]. Injectable drugs are often demanded because they are perceived to be a more effective treatment [28,29]. These practices were found to be imbedded in a sociocultural base to respond to the social expectations of the community that demanded injectable medications and wanted prompt recovery [30]. As AM rapidly eliminates asexual parasite stages and early sexual forms of falciparum malaria, it produces a rapid clinical and parasite response [31].

In the district of Bata most of the children were not prescribed ASAQ as treatment, even when the antimalarial was available at the health facility’s pharmacy. The low use of the first-line recommended treatment might be related to the perception about the side effects of this ACT [32]. These predispositions about ASAQ might also explain the frequent use of AL in Bata as first-line treatment for uncomplicated malaria. Beliefs by healthcare workers about a medication could greatly influence the prescription patterns [33]. The highest prescription of AL versus ASAQ is supporting the necessity to discuss changing the MNTG and establishing AL as first-line treatment, if the ongoing study about therapeutic efficacy of AL versus ASAQ would allow it.

Moreover, some of the prescriptions found in this study have not been described elsewhere. The frequent prescription of injectable AM plus a single dose of oral SP is a combination that is not included in the MNTG or in any WHO anti-malarial treatment recommendations. The inadequate use of injectable AM as first-line treatment could promote the development of artemisinin derivative resistance and lead to ACT treatment failure [34,35]. While SP is the medication reserved for the Intermittent Preventive Treatment (IPT) of malaria in pregnant women in Equatorial Guinea [9], and its frequent use as treatment may compromise the efficacy of this important preventive therapy [36,37].

According to the MNTG, intravenous artesunate is recommended for the management of severe malaria until the patient regains consciousness, then full ASAQ therapy should be administered. Prescribing artemether injection has not justification in cases of uncomplicated malaria even when first-line treatment was not available. Receiving injectable AM as first-line treatment in the Bata District was found associated, in previous studies, with symptoms like nausea and weakness, but not with any other important symptom related to the severity of the patients [11].

Previous studies showed how inappropriate prescription practices are also extended to the private health sector and pharmaceutical sellers [10]. This situation is generating a treatment market that seems to be based more on profitability than in public health criteria [38], especially when artemether is more expensive than AS/AQ in Bata district [11]. There is an urgent need to improve malaria treatment practices in the public and private health sectors in Equatorial Guinea.

The MINSABS should ensure that first-line ACT is delivered on time to all health facilities, which does not happen in the continental region of Equatorial Guinea since 2012 [21]. The pharmacy managers of the public health facilities have to purchase their own antimalarial drugs from private distributors, and consequently they could acquire the ACT desired. Therefore, the presence or absence of ASAQ at the health facility pharmacies could also be related to the preferences of practitioners based on prescriptions. Only two health facilities had ASAQ in stock at the time of the study, but most of the children diagnosed as uncomplicated malaria in those facilities were not prescribed the recommended treatment. As in other countries, the presence of stock alone does not ensure that treatment guidelines are followed [18]. More pharmacy management research is needed to understand the role of ASAQ stock-outs on practitioner prescribing behavior.

Adequate malaria case management reduces progression to severe stages, and therefore affects the morbidity and mortality rates [39]. The availability of malaria diagnostic tools in public health facilities was higher in Equatorial Guinea than other countries in the region, [17]; however, presumptive diagnosis, even when diagnostic tools were available, still occurs in the Bata District in almost 20% of cases. Treatment under a presumptive diagnosis in an endemic area implies a lower diagnostic specificity and administration of unnecessary antimalarial therapies [40], but also underdiagnoses of other severe diseases that present with fevers [41]. Even when the presumptive diagnosis is lower in Equatorial Guinea than other countries in the region [4,17], it is still necessary to reinforce the importance of administering antimalarial therapies with diagnostic confirmation.

Despite knowledge not always implies better dosage and therapeutic management [30], in the Bata District practitioners who knew the therapeutic guide were three times more likely to prescribe recommended treatment. In addition, there is an important association between good practices and the compliance with first-line treatment. Practitioners who rely on parasite confirmation for malaria diagnosis were almost six times more likely to prescribe MNTG treatment. The ACTs and new therapeutic guide were introduced in the continental region of Equatorial Guinea in 2008. Since then, no additional training has been offered to healthcare professionals. There is an urgent need to implement new healthcare worker training in the management of malaria cases, but also post-training follow-up and structured supportive supervision that could guarantee changes in practice [17,42].

The main limitation of this study was the possible Hawthorne effect, resulting in a modification of behavior on the part of the prescribers due to the presence of the research team during the clinic consultation and therefore their compliance with the national protocol could be even lower. Finally, this was a cross-sectional study in the Bata District, so the results may not be generalizable to the rest of the country, even when low compliance with first-line malaria treatment throughout the country is one of the NPMC main concerns.

## Conclusions

This study identified the factors associated with the low compliance with the first-line malaria treatment by the public healthcare facilities of Bata District of Equatorial Guinea. According with results, it seems necessary to improve case malaria management of children with uncomplicated malaria starting by reinforcing the use of Malaria National Therapeutic Guide and informing about the danger of using artemisinin monotherapy. Furthermore, it is crucial to provide recommended first-line treatment to the pharmacies of all public health facilities to ensure access to this treatment and avoid possible free choices in the procurement of antimalarial drugs. It seems also necessary to strength the diagnostic capacity of practitioners, their management of malaria symptoms, and the relevance to base their malaria diagnosis on parasite confirmation.
